# Evaluation of calcium β-hydroxy-β-methylbutyrate on performance of *Bos indicus*-influenced cattle in a subtropical environment

**DOI:** 10.1093/jas/skaf459

**Published:** 2026-01-06

**Authors:** Rodrigo D L Pacheco, Jéssica O Gusmão, Alex S Maia, Pedro H P Terêncio, João P S T Bastos, Ricardo Ávila, Bradley J Johnson, Victor P C Campanelli, Vinícius F C Fonsêca, Gustavo A B Moura, Rafael Da C Cervieri, John C Fuller Jr.

**Affiliations:** Agro-Pastoril Paschoal Campanelli, Research Center, Altair, São Paulo, 15430-000, Brazil; Agro-Pastoril Paschoal Campanelli, Research Center, Altair, São Paulo, 15430-000, Brazil; Innovation in Sustainability in Animal Biometeorology, Animal Biometeorology Laboratory, São Paulo State University, 14844-900, Brazil; Agro-Pastoril Paschoal Campanelli, Research Center, Altair, São Paulo, 15430-000, Brazil; Infinity Consultoria, Rua José Joaquim Francisco, Uberlândia/MG, 06541-970, Brazil; Maravedi Services and Consulting Company, São Paulo, 02441-000, Brazil; Department of Animal and Food Sciences, Texas Tech University, Lubbock, TX 79409; Agro-Pastoril Paschoal Campanelli, Research Center, Altair, São Paulo, 15430-000, Brazil; Innovation in Sustainability in Animal Biometeorology, Animal Biometeorology Laboratory, São Paulo State University, 14844-900, Brazil; Innovation in Sustainability in Animal Biometeorology, Animal Biometeorology Laboratory, São Paulo State University, 14844-900, Brazil; Nutribeef Consulting, Botucatu, São Paulo, 18609-660, Brazil; Metabolic Technologies, Missoula, MT 59802

**Keywords:** *Bos indicus*, CaHMB, cattle, performance, shade, subtropical environment

## Abstract

β-Hydroxy-β-methylbutyrate (HMB), a metabolite of leucine, increases both protein synthesis and lipolysis, immune function, and overall health in both humans and animals. Previous cattle studies have shown that HMB, fed as calcium HMB (CaHMB), can minimize stress responses and improve meat quality. The objective of the present study was to investigate the effect of feeding CaHMB in a subtropical feedlot on performance of Nellore (*Bos indicus*) bulls. A total of 3,520 bulls (64 pens; 55 animals per pen) were studied with four levels of CaHMB (0 [control, CON], 3, 5, or 7 g per head per day) and half the pens had shade access (SH), and half did not in a split-plot experimental design. Diets containing CaHMB were fed during three phases of the study, adaptation, growth, and finishing. There were no CaHMB × SH interactions observed for any variable measured. However, adjusted final body weight (FBW) tended to increase in CaHMB-treated bulls up to 5.8 kg in bulls fed 3 g per day (linear *P *< 0.07). Additionally, adjusted average daily gain (ADG) and carcass ADG were also increased with 3 g per day CaHMB by 3.2% and 2.6%, respectively, when compared with CON (linear *P *< 0.04). Bulls fed CaHMB tended to have increased hot carcass weight (HCW) by up to 3.4 kg in bulls fed 3 g CaHMB per day and a 0.36% increase in dressing percentage (DP) (linear *P *< 0.07 and 0.09, respectively). No differences were observed in dry matter intake (DMI) during any of the feeding periods. No significant differences were observed in gain:feed. Of major importance to the subtropical environment, 34% of the days were considered hot to very hot with the comfort level (InCI) >0.45 on a 0 to 1.0 scale. In conclusion, supplementing *Bos indicus* bulls with CaHMB resulted in improved growth performance and carcass yield.

## Teaser

Improving beef cattle feedlot performance is important to facilitate the increasing world demand for beef. The leucine metabolite, β-hydroxy-β-methylbutyrate (HMB), has shown potential to improve feedlot performance in *Bos taurus*bred cattle when fed as the calcium salt (CaHMB). Data from this trial indicated that the use of CaHMB is justified for cattle in tropical regions.

## Graphical abstract

**Graphical Abstract:**
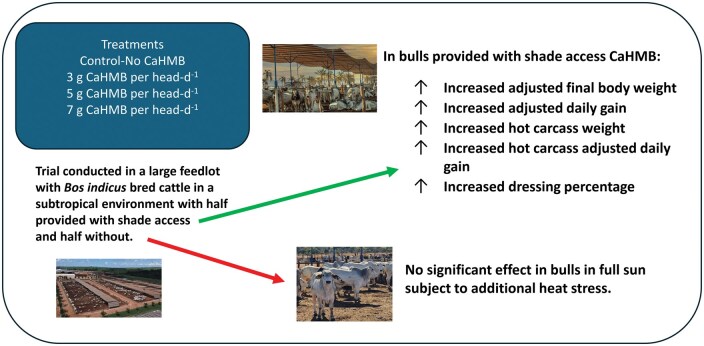

## Introduction

The leucine metabolite, β-hydroxy-β-methylbutyrate (HMB), has been shown to have a positive effect on protein and lipid metabolism, immune function, and overall health in both humans and animals (Nissen and Abumrad, 1997; Nissen et al., 2000; Szczesniak et al., 2015; Wilkinson et al., 2018). The calcium salt of HMB (CaHMB) is commonly used as a dietary supplement for performance enhancement in exercising individuals (Kerksick et al., 2018; Gepner et al., 2019) and for maintaining muscle mass and function in the elderly (Baier et al., 2009; Deutz et al., 2013; Berton et al., 2015).

Additionally, HMB may improve health and immune function in sheep (Nissen et al., 1990) and cattle (Van Koevering et al., 1993; Wójcik et al., 2013, 2014, 2020). Van Koevering et al. (1993) observed a trend for improved average daily gain and numerical decreases in calf morbidity, calves needing retreatment, number of treatment days, and cost of treatment in shipping stressed calves. Talleyrand et al. (1994) fed CaHMB to calves for 5 wk before inducing stress via adrenocorticotropic hormone (ACTH) injection and found CaHMB lowered serum cortisol levels and increased blood t- and b-cells. Recent studies have shown that feeding CaHMB stimulates non-specific cell-mediated immunity, improves phagocytic function, and increases gamma globulin level and lysozyme activity (Wójcik et al., 2013, 2014, 2020).

Cattle fed CaHMB during finishing showed a repartitioning of intramuscular to intermuscular fat resulting in increased marbling and higher yield grades (Van Koevering et al., 1994). The use of CaHMB in diets for 82 d during finishing increased average daily gain, dry matter intake and improved gain:feed. In addition, subcutaneous fat thickness was reduced due to increased lipid oxidation, which improved carcass yield (Van Koevering et al., 1994).

To date, no large-scale studies with CaHMB in either a subtropical environment or for the Nellore breed (*Bos indicus*) have been conducted. The Nellore breed has higher protein requirements compared to *Bos taurus* breeds (Filho et al., 2016), and thus CaHMB could be advantageous in optimizing protein utilization in Nellore cattle. In poultry, supplementing CaHMB has been shown to have multiple effects on growth and protein metabolism which results in increased carcass and meat yields (Nissen et al., 1994; Ostaszewski et al., 2000; Qiao et al., 2013). Less is known about the effect of supplementation in ruminants. Additionally, conducting the study in a subtropical environment with prolonged exposure to high temperature, humidity, and solar radiation may impair digestive function and reduce feed intake (Maia et al., 2023; Scerri et al., 2023). Other studies in cattle have shown that dosages from 4 to over 6 g/head-d^−1^ could be beneficial in stressed animals (Van Koevering et al., 1993; Talleyrand, 1995), Therefore, in the present study we utilized the 3 g/d dosage as shown in the Van Koevering et al. (1994) study in *Bos taurus* type cattle and also used higher doses of 5 and 7 g/d, which may be more effective in decreasing any heat stress response, which may be seen in the Nellore cattle in the subtropical environment.

Our hypothesis was that bulls supplemented with CaHMB could have improved growth performance and carcass traits. Therefore, the objective of the current study was to evaluate the effect of different dosages of CaHMB dietary supplementation on growth performance and carcass traits in the commercial feedlot in *Bos indicus* (Nellore) cattle, both with and without partial shade, under subtropical conditions.

## Materials and Methods

All animal care and experimental procedures were approved by the Universidade Estadual Paulista Faculdade de Ciências Agrárias e Veterinárias (São Paulo, Brazil) animal use and ethics committee protocol approval (UNESP; Protocol number: 016339/19) and followed the Guide for the Care and Use of Agricultural Animals in Research and Teaching (Tucker et al., 2020).

### Pre-experimental procedure

A total of 3,737 animals predominantly *Bos indicus* intact males (predominantly Nellore) with an average body weight (BW) of 375 ± 34 kg (mean ± SD), approximately 24 month old, were received from 16 different stocker ranches with an average transportation distance of 693 km (minimum 50, maximum 910 km) at the Campanelli Innovation Center (CIC, Altair, São Paulo, Brazil). Bulls were allocated by body weight (BW within a 40 to 60 kg range), into seven 12-hectare *Cynodon dactylon* grass paddocks equipped with feed bunks. A pre-experimental maintenance diet (Table 1), without CaHMB in the diet, was offered *ad libitum* and fed for between 4 and 10 d as groups arrived. The diet was formulated to help re-establish the ruminal environment and equalize physiological conditions and fed states of all bulls before the experimental diets were offered.

**Table 1.** Diet compositions

	Diets^1^			
Item	Pre-exp	Adaptation	Growing	Finishing
**Ingredient (%, DM basis)**				
** Corn silage**	27.25	45.36	31.70	19.35
**Wet sugar cane bagasse**	19.74	–	–	–
**Pelleted citrus pulp**	–	12.47	19.58	28.22
**Reconstituted sorghum silage**	–	12.47	19.58	28.22
** Soybean molasses**	12.04	–	–	–
** Cottonseed cake**	8.74	17.40	17.34	10.68
**Pelleted soybean hulls**	26.14	6.24	5.78	7.49
** Corn steep liquor**	2.86	3.06	3.03	3.04
**Dry trace mineral supplement^2^**	3.23	3.00	3.00	3.00
**Diet composition**				
** DM, %**	54.0	50.0	56.0	62.0
** CP, %**	14.0	15.5	15.5	14.6
** EE, %**	2.40	2.90	3.10	2.60
** NDF, %**	47.7	36.7	32.8	27.9
** NFC, %**	29.2	42.8	46.6	52.8
** NEm, Mcal/kg**	1.49	1.79	1.81	1.87
** NEg, Mcal/kg**	0.90	1.16	1.18	1.23

1 The pre-experiment diet was fed upon arrival of the bulls for between 4 and 10 d before initial weighing and feeding of the dietary treatments. The adaptation diet was fed for 17 d, the growing diet for 30 d, and the finishing diet from 67 to 88 d, according to block IBW.

2 90% of CP (urea, 32% inclusion), calcium 120 g/kg, phosphorus 22.0 g/kg, cobalt 20.0 mg/kg, copper 270.0 mg/kg, sodium 40.0 g/kg, sulphur 30.0 g/kg, iodine 20.0 mg/kg, magnesium 10.0 g/kg, manganese 500.0 mg/kg, selenium 10.0 mg/kg, zinc 1,800.0 mg/kg, vitamin A 70,000 IU/kg, vitamin D_3_ 13,000 IU/kg, vitamin E 550 IU/kg, Monensin 834 mg/kg.

After the adjustment feeding period, the bulls were ear tagged, dewormed with an oral drench of 1 mL per 20 kg of body weight (BW) of 10% fenbendazole (Panacur, MSD Saúde Animal, São Paulo, São Paulo, Brazil), and vaccinated against bovine respiratory disease (2 mL in subcutaneous injection; Bovilis, MSD Saúde Animal, São Paulo, Brazil). Additionally, all bulls were vaccinated against clostridia (5 mL subcutaneous injection; Poli-Star, Valée S/A, Montes Claros, Minas Gerais, Brazil). At the end of this process, the bulls were weighed, stratified by body weight to pens, and fed the pre-experimental diet for at least 4 d.

For the start of the experimental period, the bulls were again weighed after 16-h feed and water withdrawal to determine the initial body weight (IBW). Bulls with two standard deviations above or below the mean were excluded from the experiment. Also, those that were assessed as visually ill, lame or were judged breed uncharacterized or having a crossbreed characteristic (e.g., a white coated Zebu without a hump which indicates some percentage of Britannic or continental breeding) by trained personnel at the sorting chute were also excluded to reduce cattle breed variability. Following weighing, using a lottery algorithm macro with random number function in Microsoft Excel 2011 (Microsoft Corporation, Redmont, Washington, USA), bulls were assigned to their respective experimental pen, with a similar mean weight and coefficient of variation in every pen within an experimental block (body weight grouping). Selected bulls for study enrollment were put on the study over eight different start dates over a duration of approximately 21 d. Body weight for each start date was as follows: 345 ± 14 kg (block 1); 339 ± 15 (block 2); 375 ± 10 kg (block 3); 376 ± 19 kg (block 4); 391 ± 18 kg (block 5); 368 ± 15 kg (block 6); 419 ± 22 kg (block 7); and 398 ± 26 kg (block 8). A total of 3,520 *Bos indicus* (Nellore) bulls were selected for the study.

### Experimental design and pen assignment

The study was an unreplicated split-plot (shade [SH] vs. no shade [SUN]) with a blocked distribution of dietary treatments within each split-plot, SH vs. SUN. Four dietary treatment levels of CaHMB were used and the bulls were assigned to one of eight replications with SH or eight replications in SUN. The bulls were allocated to open dry lot pens (50 × 15 m, 13 m^2^/bull) with 0.27 m of linear bunk space/bull and a water trough (3.0 m length × 0.8 m height × 0.25 m width). The pens with SH access had 2.4 m^2^ shade/bull. In pens with SH access, shades were positioned 5 m from the ground with 18-degree displacement in northeast to southwest direction. A map of penning design is in the supplementary materials Fig. S1.

### Calcium β-hydroxy-β-methylbutyrate (CaHMB) treatments

Four levels of CaHMB (TSI Group Co., Ltd, Jiangyin China) were supplemented during the study: control diet 0 (HMB0, CON); 3 g/d (0.3 g/kg of dry matter (DM), HMB3); 5 g/d (0.5 g/kg of DM, HMB5) and 7 g/d (0.7 g/kg of DM, HMB7). Previously in a study by Van Koevering et al. (1994) 3 g/head-d^−1^ were fed. However, results were inconsistent and dependent upon length of time the CaHMB was fed. The CaHMB treatments were offered in the mineral premix, and kaolin was used as inert material to maintain target nutrient levels in all diets. Mineral premixes were sampled at two timepoints during the experiments and analyzed for CaHMB (Heartland Assays, Ames, IA, USA). The average of the two analyses showed the 0-, 3-, 5-, and 7-g CaHMB per day diets supplied 0, 2.96, 5.11, and 8.03 g CaHMB per day on average.

### Feeding and health management

The feeding program consisted of three diets: adaptation, growing, and finishing. Cattle on all treatment diets received monensin at 25 mg/kg of DM during the experiment. The adaptation diet was fed for 17 d, the growing diet for 30 d, and the finishing diet from 67 to 88 d, according to IBW. Nutrient levels and diet compositions are presented in Table 1. Bulls were fed twice daily at 0730 and 1400 h, with bunk score calls recorded daily at 0645 h, following a modification of the method of Pritchard and Bruns (2003) to achieve feed refusals of approximately 1% to 2% of feed offered. Feed delivery was equally divided between morning and afternoon offers. Individual pen feed refusals were weighed every morning before the first feed delivery with a ± 0.1 kg precision electronic scale (Alfa Instruments, Samel-2CF, São Paulo, São Paulo). This procedure was adopted to ensure that the bunks always contained feed. Fresh feed was then delivered using a truck-mounted mixer (Brutale, Model MTB-120CM2 32-m^3^ capacity, São Carlos, São Paulo, Brazil) equipped with electronic scale (±1 kg precision). The scale was calibrated weekly during the experiments. The mixer was emptied and cleaned by brushing or rinsing as needed before every diet change (four times in the morning and four times in the afternoon). The CON diet was fed first at each time.

One trained person checked bulls for signs of disease twice daily. If needed, bulls were treated with flofernicol and flunixin meglumine (intramuscular injection, 1 mL per 7.5 kg of BW, Resflor Gold^®^, MSD Saúde Animal, São Paulo, São Paulo, Brazil) for pulmonary issues or using tildipirosin (intramuscular injection, 1 mL per 45 kg of BW, Zuprevo^®^, MSD Saúde Animal, São Paulo, São Paulo, Brazil) when hoof issues (hoof rot and laminitis) were detected. For both disease conditions, a second treatment was given if necessary, and the bull was removed from the experiment if recovery was not indicated.

### Performance and carcass variables

Average dry matter intake (DMI) was calculated daily by the difference of offered feed and refusals. On the last day of the experimental feeding period, to obtain a final body weight (FBW) measurement, bulls were withheld from feed and water for 16 h. Performance data such as average daily gain (ADG) and gain:feed were calculated based on shrunk IBW and shrunk FBW, using the mean DMI of entire experimental period. At the end of the experiment, bulls were harvested at a commercial packing plant located 330 km from the feedlot. Bulls were harvested on eight separate dates, according to weight blocks (heavier to lighter). Harvest weight was defined when bulls reached an average of 560 kg of shrunk FBW.

Hot carcass weight (HCW) was obtained after evisceration and removal of kidney, pelvic, and heart fat. Dressing percentage (DP) was calculated as the ratio of HCW to shrunk FBW. In order to more accurately compare the lighter and heavier cattle as different days of feeding were necessary to reach harvest FBW, average DP (57.86%) of all bulls was used to estimate adjusted FBW (FBW_adj_=HCW/DP), adjusted ADG (ADG_adj_=(FBW_adj_ − IBW)/Days on feed), and adjusted gain:feed (gain:feed_adj_=ADG_adj_/daily DMI) (de Melo et al., 2019).

### Chemical analyses

Diet dry matter (DM) adjustments of feed ingredients were conducted twice daily throughout the experiment using a hot air ventilated oven in which the feed samples were dried at 120 °C for one hour (Air Fryer, Model AF-30-I, Mondial, 3.5 L capacity, 1500 W, Conceição do Jacuípe, BA, Brazil). In a similar manner, total mixed diet and feed refusals were collected twice and once daily, respectively. For both variables, diet composite samples (based on equal amounts of samples per pen) were generated and dried at 105 °C (Teknal, model TE-394/3-MP, Piracicaba, São Paulo, Brazil; method 930.15, AOAC, 2000) for 12 h to determine DM.

Samples of the diets were collected weekly during feed delivery at three times during delivery (first, middle, and last pens), composited, and sampled for chemical analyses (Table 1). All samples were dried at 55 °C in a forced-air oven for 72 h for DM determination. Dried samples were ground in a Wiley-type mill (1-mm screen, MA-680, Marconi Ltda, Piracicaba, São Paulo, Brazil) and analyzed for ash (method 924.05 [AOAC, 1995]), NDF (Van Soest et al., 1991), CP (AOAC, 2012) and EE (method 920.85 [AOAC, 1986]). The NFC was estimated according to the following equation: NFC (%) = 100% − (% NDF + % CP + % EE + % ash), according to Mertens (­Mertens, 1997).

### Meteorological data collection

Meteorological data were collected as previously described (Maia et al., 2023). Briefly, solar irradiance, ultraviolet solar radiation, air temperature, black-globe temperature in the sun, relative humidity, and wind speed were recorded using a portable weather station (WS-18 model 110, Nova Lynk, Auburn, CA, USA) placed near the pens. An index of bull comfort (InCI) was calculated as described in previously published studies (Maia et al., 2023; Pacheco et al., 2023). Briefly, the developed index of bull comfort uses a combination of the six weather variables with respiration rate and water intake of the bulls. This index is a better estimate of animal comfort in the tropics because solar radiation is directly considered with this model in determining the comfort or heat stress level.

### Statistical analyses

Data were first checked for normality by analysis of residuals (Royston, 1982), homoscedasticity (Levene, 1960), and for outliers (Lund, 1975). Feedlot performance and carcass characteristics were analyzed using the PROC GLIMMIX procedure of the Statistical Analysis System (SAS) (SAS/STAT^®^ 9.3 User’s Guide. SAS Institute, Inc., Cary, NC, 2011) as an unreplicated split-plot with either SH or SUN. Dietary levels of CaHMB were blocked and replicated within each split-plot (SH or SUN). Pens were considered as the experimental unit in analyses. The model used was effect = split-plot block treatment split-plot * treatment where split-plot is SH or SUN, block is treatment blocking within split-plot, and split-plot * treatment interaction is to account for any confounding effects due to facility differences (SH vs. SUN). The random effect in the model was treatment (pen). Contrasts were performed to evaluate linear and quadratic dose responses. In dose ranging studies, quadratic treatment significance can indicate an optimal dosage has been reached or exceeded (Hales et al., 2017). Dry matter intakes were measured daily, and the Generalized Additive Models Procedure (GAM) was used to predict DMI. The model used was DMI = split-plot treatment split-plot * treatment Phase (split-plot * treatment) InCI (split-plot * treatment) DOF IBW, where split-plot is SH or SUN, InCI is comfort level, and DOF is days on feed. Spline smooth functions were applied, and data are presented as means and confidence interval at 95%. For health issues, incidences (sum of all health issues) were analyzed using PROC LOGISTIC. The model was incidence = split-plot block treatment split-plot * treatment where split-plot is SH or SUN and split-plot * treatment interaction is to account for any confounding of treatment effect due to the split-plot design. Significant differences were determined by Chi square tests comparing the control group with each CaHMB level group. Differences were considered significant when *P *≤ 0.05 and regarded as tendencies when *P > *0.05 and *P *≤ 0.10.

## Results

### Feedlot performance

There were no CaHMB × SH interactions observed for any of the feedlot performance variables measured (Table 2). A trend was observed (linear effect, *P *< 0.07) for adjusted FBW, in which HMB3-treated bulls showed the greatest improvement and were 5.8 kg heavier when shade was provided. The HMB3-treated group also had a 3.2% increase in adjusted ADG (linear effect *P *< 0.04).

**Table 2.** Feedlot performance of Nellore bulls (*Bos indicus*) supplemented with calcium β-hydroxy-β-methylbutyrate (CaHMB) with half of the pens provided with shade area while the other half had no shade area^1^

	Structure														
	Sun				Shade										
	Dietary treatment^2^										*P*-value				
	CON	HMB3	HMB5	HMB7		CON	HMB3	HMB5	HMB7	SEM	Diet	Shade	Diet × Shade	Linear^3^	Quadratic^3^
**IBW, kg**	376	376	377	377		376	376	376	377	0.13	0.06	0.76	0.50	0.02	0.27
**FBW, kg**	575	574	576	574		575	577	574	573	1.60	0.85	0.98	0.74	0.48	0.61
**ADG, kg/d**	1.560	1.550	1.561	1.545		1.556	1.578	1.547	1.540	0.009	0.67	0.93	0.65	0.33	0.46
**Gain:feed**	0.1567	0.1556	0.1579	0.1564		0.1580	0.1581	0.1570	0.1594	0.001	0.97	0.31	0.78	0.75	0.75
**FBW_adj_, kg**	576.2	573.7	574.8	572.3		573.9	579.7	575.3	571.2	0.997	0.11	0.59	0.18	0.07	0.09
**ADG_adj_, kg/d**	1.567	1.545	1.554	1.532		1.548	1.598	1.560	1.523	0.008	0.04	0.49	0.11	0.04	0.05
**Gain:feed_adj_**	0.1574	0.1551	0.1573	0.1549		0.1575	0.1599	0.1581	0.1575	0.001	0.85	0.12	0.62	0.55	0.56

1 Results of all animals, *n* = 16 pens of 55 bulls per diet (8 with shade and 8 in full sun).

2 Dietary treatments were CON, control or no CaHMB; HMB3, HMB5, HMB7 indicating 3, 5, or 7 g CaHMB per head per day during the entire study.

3 Linear and Quadratic effects of the increasing treatment dosages (0, 3, 5, and 7 g/d) were determined using contrast statements.

No SH vs. SUN interactions with diet were found for dry matter intake (Table 3). Dry matter intake was not different among all treatment conditions, except for a small but significant difference between SH and SUN when the growing diet was fed (*P *< 0.04). Contributing to this significant finding was the actual DMI of the HMB7 dietary treatment group with shade. Overall, this group had the numerically lowest DMI in total and the lowest DMI during the growing diet feeding. However, there was not a significant linear or quadratic effect of dietary treatment during this period.

**Table 3.** Dry matter intake of Nellore bulls (*Bos indicus)* supplemented with calcium β-hydroxy-β-methylbutyrate (CaHMB) with half of the pens provided with shade area while the other half had no shade area^1^

	Structure														
	Sun				Shade										
	Dietary treatment^2^										*P*-value				
Period	CON	HMB3	HMB5	HMB7		CON	HMB3	HMB5	HMB7	SEM	Diet	Shade	Diet × Shade	Linear^3^	Quadratic^3^
**Adaptation, kg/d**	9.78	9.77	9.88	9.67		9.82	9.75	9.83	9.67	0.107	0.39	0.90	0.98	0.39	0.33
**Growing, kg/d**	11.26	11.14	11.21	11.15		10.92	11.13	11.09	10.85	0.182	0.66	0.04	0.56	0.51	0.31
**Finishing, kg/d**	9.51	9.57	9.42	9.50		9.42	9.57	9.42	9.22	0.136	0.47	0.34	0.72	0.28	0.41
**Total study, kg/d**	9.97	9.98	9.91	9.92		9.84	9.97	9.88	9.68	0.121	0.55	0.23	0.76	0.30	0.34

1 Results of all animals, *n* = 16 pens of 55 bulls per diet (8 with shade and 8 in full sun).

2 Dietary treatments were CON, control or no CaHMB; HMB3, HMB5, HMB7 indicating 3, 5, or 7 g CaHMB per head per day during the entire study.

3 Linear and Quadratic effects of the increasing treatment dosages (0, 3, 5, and 7 g/d) were determined using contrast statements.

Carcass characteristics are shown in Table 4 and a trend for increased carcass weight was observed (linear *P* < 0.07 and quadratic *P* < 0.09). The HMB3-treated bulls had a 3.4 kg increase in hot carcass weights compared with the CON in the SH. Additionally, there was a significant diet effect on carcass daily gain with the HMB3-treated bulls having a daily gain 2.6% greater than the CON with SH (diet *P *< 0.04, linear effect *P *< 0.04). Dressing percentage tended to increase with dietary treatment (diet *P *< 0.10) and this effect tended to be more quadratic (*P *< 0.06) than linear (*P *< 0.09) indicating the HMB3-treated bulls with SH had the greatest DP while the HMB7-treated bulls with SH had the lowest DP.

**Table 4.** Carcass characteristics of Nellore bulls (*Bos indicus*) supplemented with calcium β-hydroxy-β-methylbutyrate (CaHMB) with half of the pens provided with shade area while the other half had no shade area^1^

	Structure														
	Sun				Shade										
	Dietary Treatment^2^										*P*-value				
	CON	HMB3	HMB5	HMB7		CON	HMB3	HMB5	HMB7	SEM	Diet	Shade	Diet × Shade	Linear^3^	Quadratic^3^
**HCW, kg**	332.6	332.1	332.8	331.3		332.2	335.6	333.1	330.7	1.16	0.11	0.59	0.18	0.07	0.09
**Carcass daily gain, kg/d**	1.142	1.129	1.134	1.121		1.131	1.160	1.138	1.116	0.009	0.04	0.48	0.11	0.04	0.04
**Carcass gain: feed**	0.1145	0.1131	0.1146	0.1134		0.1150	0.1160	0.1151	0.1153	0.009	0.97	0.12	0.75	0.81	0.84
**Dressing percentage, %**	57.98	57.84	57.80	57.71		57.79	58.15	58.04	57.68	0.12	0.10	0.35	0.16	0.09	0.06
**pH**	5.75	5.77	5.75	5.76		5.76	5.75	5.74	5.78	0.01	0.50	0.80	0.30	0.60	0.42

1 Results of all animals, *n* = 16 pens of 55 bulls per diet (8 with shade and 8 in full sun).

2 Dietary treatments were CON, control or no CaHMB; HMB3, HMB5, HMB7 indicating 3, 5, or 7 g CaHMB per head per day during the entire study.

3 Linear and Quadratic effects of the increasing treatment dosages (0, 3, 5, and 7 g/d) were determined using contrast statements.

### Animal health

Morbidity and animal treatment for health issues were not significant between CON bulls and CaHMB-treated bulls. The incidences were so low that SUN and SH had to be combined for a valid analysis. Therefore, no differences between SUN and SH were detected. Incidences of morbidity were either respiratory or hoof problems during the study and the numbers of incidences were 25, 33, 23, and 27 for CON, HMB3, HMB5, and HMB7 (Chi Square, *P *< 0.15, 0.21, and 0.67 for HMB3, HMB5, and HMB7 vs. CON, respectively). A breakout by morbidity cause and bulls removed from the study is shown in Supplementary Table 1.

### Meteorological data

**Figure 1.**
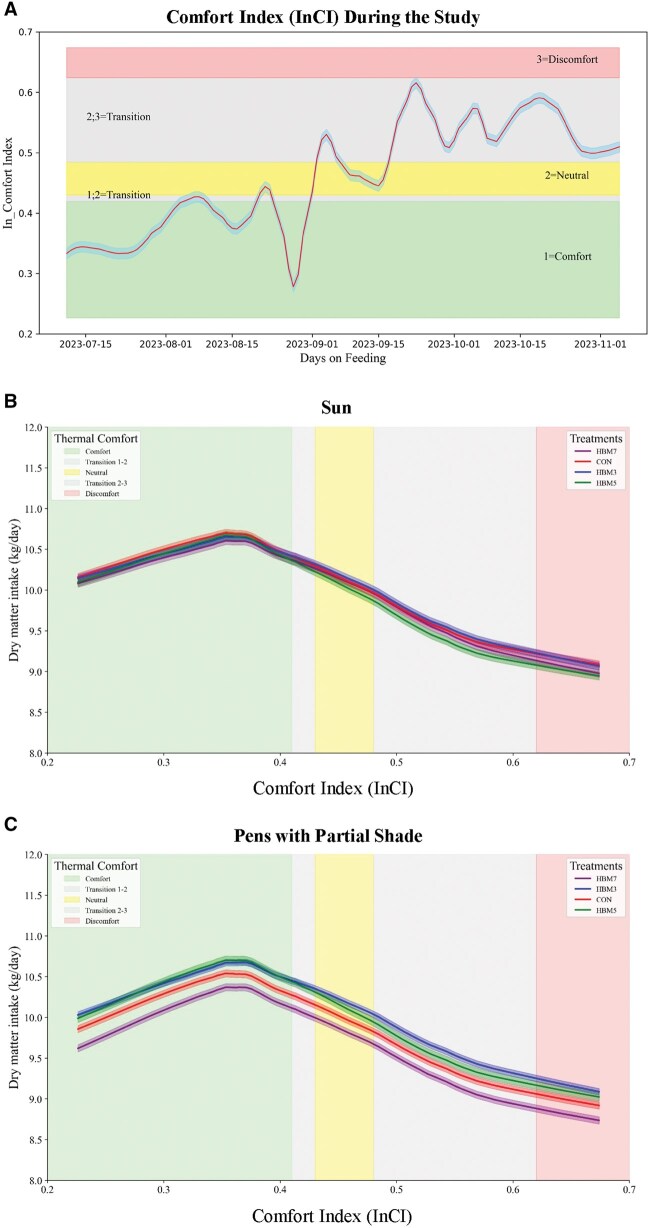
Comfort index (InCI) level by day of the study (A). Dry matter intake (DMI) by InCI level for pens in sun without any shade (B). DMI by InCI level for pens with shade access (C). CON is control or no CaHMB and HMB3, HMB5, and HMB7 are 3, 5, and 7 g CaHMB per head per day, respectively.

Analysis of meteorological data showed approximately 34% of the days were classified as above InCI level 2 and during the last half of the study, many days were very hot and near the high end of the InCI range (Figure 1A). Comfort, InCI level, affected DMI as shown in Figure 1B and C. In general, as InCI level increases, feed intake decreases. Without provided shade there were no differences in DMI; however, GAM analysis of bulls with provided shade access showed HMB3 and HMB5 did have increased feed intake during the neutral comfort phase, which continued into the higher InCI measurements towards the end of the study.

## Discussion

Information is lacking on the effects of CaHMB, therefore, we have conducted the first large scale feedlot study conducted in a subtropical zone investigating the effects of CaHMB upon *Bos indicus* bred cattle. In this study, we have shown that overall yield is increased in Nellore bulls supplemented with CaHMB. The primary effect was an improvement in average daily gain with three grams to CaHMB fed per head per day and tended to increase adjusted FBW, increased adjusted ADG, HCW, and tended to improve DP.

This study did not address the production of endogenous HMB from leucine either by the rumen microbes or the animal itself. Neither did this study determine absorption of HMB by the bulls. Metabolically HMB is produced in the body from leucine, and it has been estimated that 5%–10% of leucine may be converted to HMB for non-protein metabolic use of the amino acid leucine (Van Koevering and Nissen, 1992). Rumen production of HMB is not reported, but it is believed that most all of the HMB fed bypasses the rumen as HMB plasma levels were 80% greater in animals fed 3 g CaHMB per day 16 h post feeding when compared with cattle receiving no CaHMB (1.70 vs. 3.06 µg/mL, *P *< 0.001) (Van Koevering et al., 1994). In this same study plasma α-ketoisocaproate levels were the same whether HMB was fed or not indicating that leucine transamination, the first step in metabolism of leucine to HMB was the same in both groups. Therefore, the increased plasma HMB would be attributed to the exogenous HMB fed. Further studies into the endogenous and exogenous metabolism of HMB in the ruminant are needed to fully assess the efficacy of endogenous HMB (Van Koevering et al., 1994).

Under the perspective of a simulation of a commercial feedlot closeout, three grams of CaHMB per day demonstrated better results in terms of feedlot growth, and a trend for greater HCW was observed when shade was provided. A plausible explanation is that properly shaded feedlot facilities provide a direct effect on energy expenditure related to essential body functions, such as thermoregulation (Maia et al., 2023). Consequently, with more net energy available for growth metabolism, the greater the likelihood of observing CaHMB effects on growth, especially in subtropical regions. Moreover, during the conduct of the present experiment, 39 d were considered very hot (classified as 3 by a comfort index [InCI]) and without some shade CaHMB was likely unable to overcome this heat stress.

Dry matter intake in the current study was not significantly affected by dietary treatment nor whether shade was provided; however, bulls in shade had greater adjusted ADG. Lower initial DMI is observed in high-risk animals, such as newly weaned calves and/or animals that undergo long transportation prior to feedlot arrival. Minimization of the stress response has been shown in calves fed CaHMB through a decrease in cortisol response when challenged with adrenocorticotropic hormone (ACTH) (Talleyrand et al., 1994). Prior to the work of Talleyrand et al. (1994), Zavy et al. (1992) demonstrated that *Bos indicus* calves exhibited higher levels of cortisol than *Bos taurus* after stressors such as handling, weaning, remixing, transit, and ACTH challenge. Because CaHMB can lower stress response, it may have been possible for the improved gain without increased DMI due to the decrease in stress response from shipping and being put in the feedlot and or the heat stress during the present study. In fact, GAM analysis of feed intake compared with comfort index showed that 3 and 5 g of CaHMB helped increase DMI on the hottest days of the study if partial shade was provided.

Increased ADG coupled with trends for increased HCW and DP may indicate an increase in protein accretion in the bulls. In humans CaHMB was shown to increase protein synthesis by about 56% while decreasing protein breakdown by about 32% in younger males and researchers saw about a 3-fold increase in anabolic mTOR signaling through by p70S6K and RPS6 (Wilkinson et al., 2018). However, the researchers failed to show changes in the catabolic markers MuRF1 or MAFbx (Wilkinson et al., 2018). These findings are similar to the earlier study by Wilkinson et al. (2013) with HMB in free acid form. Earlier animal studies had shown CaHMB to be protective against attenuation of protein synthesis from catabolic factors such as proteolysis-inducing factor, angiotensin II, lipopolysaccharide, and tumor necrosis factor α (Smith et al., 2004; Eley et al., 2008). Leucine and metabolites, such as HMB, may also affect muscle protein deposition through IGF-1/mTOR pathways (Han et al., 2008; Wilkinson et al., 2018).

Growth hormone regulates the production of IGF-1 which has a potent stimulatory action on mTOR thus increasing protein synthesis. While not measured in the present study, it could be hypothesized that HMB may have had an effect on the GH/IGF-1 axis in the cattle muscle as has been demonstrated in chronic supplementation in rodents (Gerlinger-Romero et al., 2011). And in human myoblasts HMB was shown to decrease apoptosis induced by serum-starvation or staurosporine, and HMB also increased IGF-1 mRNA, markers of cell differentiation, and cell fusion through the PI3K/Akt pathway (Kornasio et al., 2009). In an injury model in rodents, HMB supplementation was shown to improve muscle recovery and decrease fibrosis in recovering muscle, also by acting through the IGF-Akt pathway (Yamada et al., 2022). Thus, IGF-1 and PI3k/Akt pathway stimulation in *Bos indicus* cattle would be important because of the need to improve muscle growth given the generally poorer quality diet that may be fed, and the toughness of the meat primarily because of muscle fiber morphology and increased connective tissue (Scheffler, 2024).

Another possible mechanism for the HMB effect on muscle hypertrophy is through satellite cell fusion with the muscle fibers. In growing animals, increasing muscle fiber diameter is the only growth mechanism as fiber numbers are established at or shortly after birth. A mechanism to enhance fiber hypertrophy is through satellite cells not fused to form the initial muscle fiber, which can fuse with the muscle fibers post birth and increase the number of nuclei, which in turn increases the muscle fibers’ ability to synthesize new protein and increase fiber hypertrophy (Alway et al., 2014). In prior studies CaHMB has been shown to increase satellite cell fusion into myofibers (Alway et al., 2013), thus a potential mechanism for increased hypertrophy and growth as seen in the present study. Further studies into this effect on bovine muscle in the feedlot are warranted.

Morbidity in the present study was low compared with average Brazilian commercial feedlots. In a study by Baptista et al. (2017) with 188,862 steers in Brazilian feedlots, overall morbidity was 7.05%. They found average mortality was 0.64%. In our study, overall treatment morbidity ranged from 2.62% to 3.76% and overall treatment mortality ranged from 0% to 0.8% with total mortality 0.38% over the study. Our morbidity and mortality incidences were lower than what is typically observed, and this may be due to the elimination of bulls that presented any evidence of illness prior to the first experimental day and by the pre-experiment feeding phase (from 4 to 10 d) that possibly allowed for an improved response to the vaccination protocol.

While this study was not designed to specifically address phase feeding, supplementing CaHMB during phases may be beneficial as CaHMB response may be affected by total days fed during the finishing period as described by Van Koevering et al. (1994). Such findings lead to the rational that phase-feeding supplementation protocols may be a more appropriate strategy to be developed. The Van Koevering et al. (1994) study also demonstrated that feeding CaHMB during finishing improved marbling and meat quality score, which results in a more desirable product for the consumer and return to the producer if meat is marketed on a quality basis. Another possible application is in dairy feedlots and/or “beef on dairy” systems, especially during the backgrounding phase, in which young, stressed animals are fed for long periods. Animals may travel long distances to the feedlot and thus endure more stress.

While the present study demonstrated positive effects of CaHMB under feedlot conditions in a subtropical climate, no growth effect on *Bos indicus* type cattle without shade during the heat stress was demonstrated. Further studies should examine the relationship between heat stress and CaHMB supplementation and the possibility that a crossbred such as *Bos indicus* × *Bos taurus* may have a different response to supplementation, as Van Koevering et al. (1994) studied a *Bos taurus* breed. Additionally, CaHMB may have influenced meat quality which was not evaluated in the present study. Therefore, given that CaHMB can enhance marbling and meat quality, further studies should examine meat quality.

## Conclusion

Supplementing *Bos indicus* bulls with CaHMB may improve growth performance and carcass yield, particularly when, even with no significant interaction, shade access is provided as this condition resulted in the greatest responses. These results may help producers decide on supplemental feeding programs for their production operations.

## Supplementary Material

skaf459_Supplementary_Data

## Data Availability

Data availability will follow a reasonable request and evaluation of the request.

## Abbreviations:

ACTH: adrenocorticotropic hormone
Adap: adaptation period
ADG: average daily gain
BW: body weight
CaHMB: calcium β-hydroxy-β-methylbutyrate
CP: crude protein
DP: dressing percentage
DM: dry matter
DMI: dry matter intake
EE: ether extract
FBW: final body weight
Fin: finishing period
Grow: growing period
HCW: hot carcass weight
HMB: β-hydroxy-β-methylbutyrate
IBW: initial body weight
mRNA: messenger ribonucleic acid
NDF: neutral detergent fiber
NE_m_: net energy maintenance
NE_g_: net energy gain
NFC: nonfiber carbohydrate
SH: shade
