# Supplementary material for: Evaluation of calcium β-hydroxy-β-methylbutyrate on performance of Bos indicus-influenced cattle in a subtropical environment
Source: J Anim Sci. 2026 Jan 6;104:skaf459. doi: 10.1093/jas/skaf459 (PMC12884845; doi:10.1093/jas/skaf459)
Supplement: skaf459_Supplementary_Data [file skaf459_supplementary_data.zip › Table S1.docx]

| Table S1. Mortality and morbidity in Nellore (*Bos indicus*) bullocks supplemented with calcium β-hydroxy-β-methylbutyrate (CaHMB). | | | | |
| --- | --- | --- | --- | --- |
|  | Diet^1^ | | | |
|  | CON | HMB3 | HMB5 | HMB7 |
| Randomized, n | 880 | 880 | 880 | 880 |
| Removed prior to treatment, n | 4 | 2 | 3 | 5 |
|  | Incidence of health problems | | | |
| Respiratory, n | 16 | 22 | 16 | 20 |
| Hoof problems, n | 9 | 11 | 7 | 7 |
|  | Removed during the treatment period | | | |
| For Respiratory, n | 1 | 1 | 0 | 1 |
| For Hoof, n | 2 | 3 | 4 | 5 |
| Mortality, n | 5 | 7 | 0 | 3 |
| Other | 0 | 1 | 1 | 2 |
| ^1^Dietary treatments were CON, control or no CaHMB; HMB3, HMB5, HMB7 indicating 3, 5, or 7 g CaHMB per head per day during the treatment period, respectively. | | | | |
