# Supplementary material for: Evaluation of calcium β-hydroxy-β-methylbutyrate on performance of Bos indicus-influenced cattle in a subtropical environment
Source: J Anim Sci. 2026 Jan 6;104:skaf459. doi: 10.1093/jas/skaf459 (PMC12884845; doi:10.1093/jas/skaf459)
Supplement: skaf459_Supplementary_Data [file skaf459_supplementary_data.zip › Figure S1.docx]

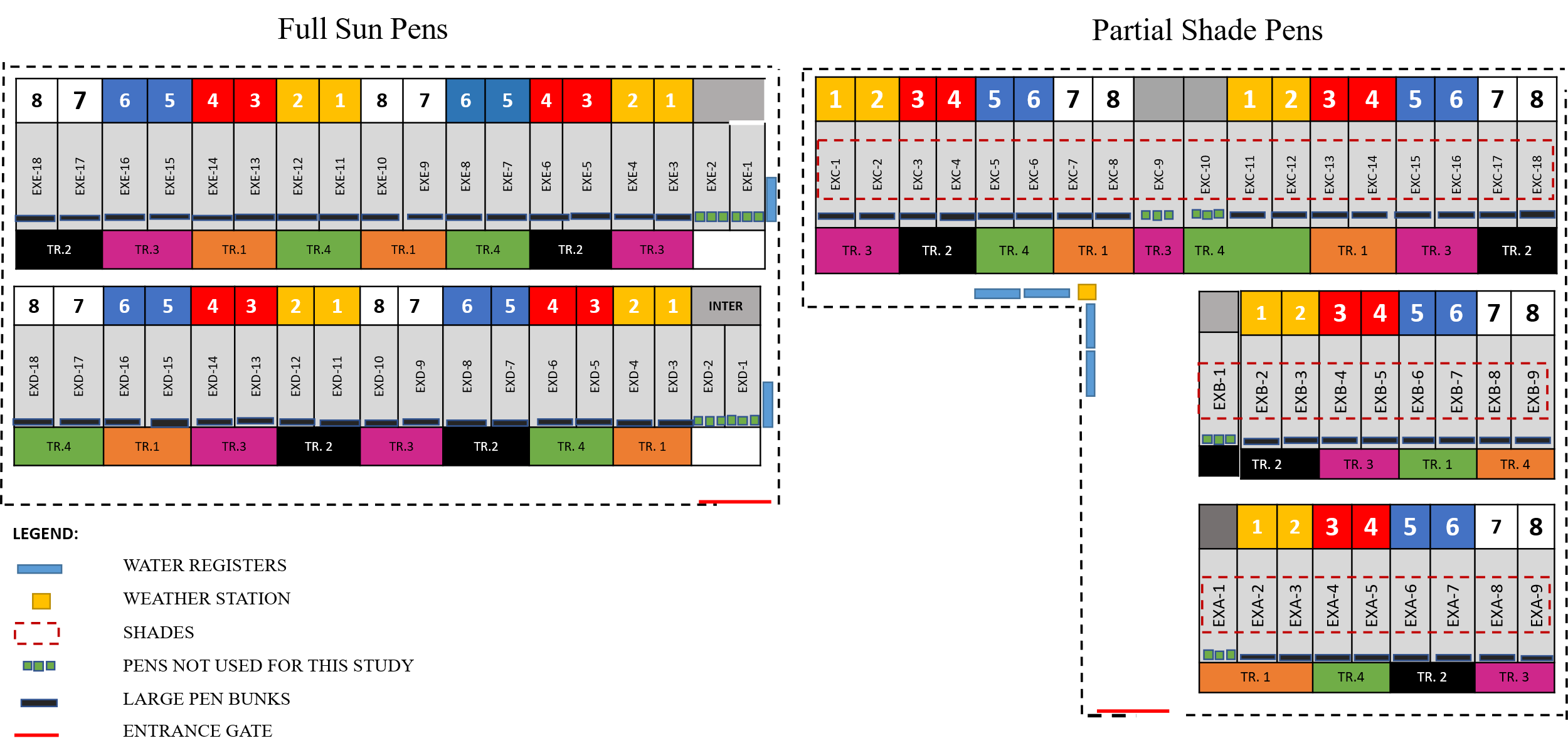


Figure S1. Penning Design. Cattle were blocked by initial body weight (IBW) into 8 blocks, BL 1-BL-8. TR.1 was control (0 g CaHMB per head/d), TR.2, TR.3, and TR.4 were 3, 5 or 7 g CaHMB per head/d, respectively.
